# Toward Dynamic Liquid Cell Scaffold: Photoreversible Ion Gels Exhibiting Light‐Induced Sol‐Gel Transitions

**DOI:** 10.1002/marc.202500909

**Published:** 2026-01-28

**Authors:** Aya Saruwatari, Yuji Kamiyama, Ryota Tamate, Jun Nakanishi, Takeshi Ueki

**Affiliations:** ^1^ Research Center for Macromolecules and Biomaterials National Institute for Materials Science Tsukuba Ibaraki Japan; ^2^ Graduate School of Life Science Hokkaido University Sapporo Hokkaido Japan; ^3^ Graduate School of Advanced Science and Engineering Waseda University Shinjuku‐ku Tokyo Japan; ^4^ Graduate School of Advanced Engineering Tokyo University of Science Katsushika‐ku Tokyo Japan

**Keywords:** azobenzene, block copolymers, gels, ionic liquids, mechanobiology, photoreversible gelation, stimuli responsive materials

## Abstract

Reversible sol–gel transitions are difficult to achieve in conventional water‐swollen hydrogels in open aqueous environments, because polymer chains dissolve or diffuse once the network disassembles. Here, we present a proof‐of‐concept to overcome this limitation by introducing a water‐immiscible and non‐cytotoxic ionic liquid (IL) phase that confines polymer networks and prevents dissolution during reversible phase transitions. We report a photoreversible ion gel that crosses the rheological boundary (tan *δ* ∼ 1) under light, enabling reversible sol–gel switching within this closed IL environment. The material integrates an ABC triblock copolymer, P(AzoAm‐*r*‐NIPAm)‐*b*‐PBuA‐*b*‐PSt, with a solvent‐quality‐tunable blend of non‐cytotoxic ILs ([P4,4,4,1][TFSI]/[P8,8,8,8][TFSI]). The photoresponsive A‐block, P(AzoAm‐*r*‐NIPAm), exhibits a polarity‐dependent solubility change with the *cis*/*trans* isomerization of azobenzene, providing a reversible light‐controlled self‐assembly. Time‐resolved rheology confirmed repeated crossings of tan *δ* = 1 under alternating UV–vis illumination at 52°C. The switching mechanism is governed by the lifetime of reversible junctions, consistent with transient network theory. In addition, hMSCs adhered to and spread on the ion gel at 37°C, indicating the cytocompatibility of the ion gel itself. This light‐programmable, water‐immiscible ion gel has the potential to provide a reversible liquid‐solid mechanical cue for next‐generation mechanobiology.

## Graphical abstract

Light‐programmable, non‐cytotoxic ion gels based on ABC triblock copolymers undergo photoreversible sol‐gel transitions, crossing the rheological boundary (tan *δ* ∼ 1). UV–vis light irradiation modulates the lifetime of reversible junctions, switching the scaffold between liquid‐like and solid (gel)‐like states. Cells adhere to and spread on ion gel in both photoisomerization states, establishing a dynamic platform for reversible liquid‐gel mechanical cues.

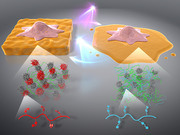

## 1 Introduction

In biological systems, mechanical environments are dynamic rather than static. During wound healing, blood coagulation, or tumor invasion, tissues transiently alter their viscoelasticity, fluidizing locally or recovering solidity, to regulate cellular processes [1, 2]. However, these transformations are generally one‐way and irreversible, driven by degradation or enzymatic reaction. In contrast, an in vitro system that could reversibly switch between liquid‐like and solid‐like states would provide an unprecedented means of applying cyclic or temporally programmed mechanical stimuli to cells, enabling the study of how cells sense dynamically fluctuating environments. Achieving such reversible sol‐gel transitions in conventional water‐swollen hydrogels is intrinsically difficult [3, 4, 5]. Although some injectable or shear‐thinning hydrogels exhibit apparent sol‐gel behavior, the process is not truly reversible under open aqueous conditions because polymer chains dissolve or diffuse into the surrounding medium once network connectivity is lost. The fundamental challenge arises from the open nature of water‐based systems; once the polymer escapes, entropy drives the system toward irreversible mixing, preventing reconstruction of the original network. Consequently, most previous studies have been confined to the modulation of stiffness within the gel regime, softening, stiffening, or cyclic variation [3, 4, 5, 6, 7, 8, 9, 10, 11, 12], without crossing the rheological boundary where the storage and loss moduli are equal (tan *δ* = 1) [13, 14].

**FIGURE 1:**
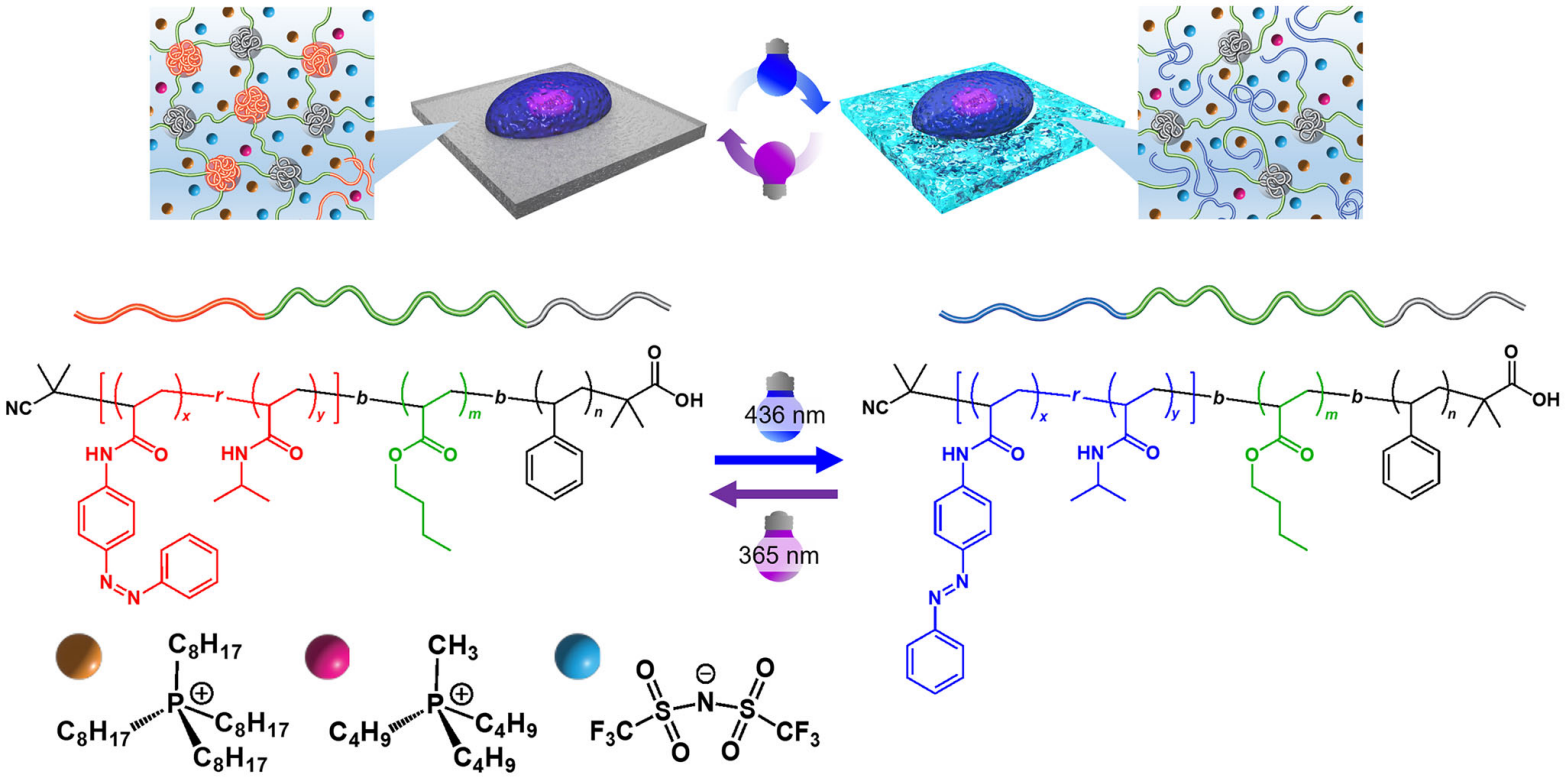
Schematic illustration of the photoreversible sol‐gel transition of the ion gel in this study. The bold curves represent the ABC‐type triblock copolymer P(AzoAm‐*r*‐NIPAm)‐*b*‐PBuA‐*b*‐PSt (ANBS), where *r* denotes random copolymerization and *b* denotes block copolymerization. Blue and red segments denote P(*trans*‐AzoAm‐*r*‐NIPAm) and P(*cis*‐AzoAm‐*r*‐NIPAm), respectively, green segments denote PBuA, and gray segments denote PSt. Upon irradiation, AzoAm undergoes reversible isomerization: UV light induces the *cis*‐form, while visible light recovers the *trans*‐form. Orange, pink, and blue spheres represent [P8,8,8,8], [P4,4,4,1], and [TFSI] ions, respectively.

To overcome this limitation, we introduce a new concept of a hydrophobic, water‐immiscible ionic liquid (IL) environment [15, 16] that confines polymers and prevents dissolution even when the network temporarily disassembles. Such ILs act as a “liquid solvent phase” that can maintain structural integrity without leakage into the aqueous culture medium. We have recently demonstrated that cells can adhere and spread at the interface between an IL and an aqueous phase, and that the interfacial viscoelasticity can be dynamically tuned by the IL composition or by electrochemical stimuli [17]. These results established that IL‐based interfaces can serve as unconventional yet stable liquid scaffolds for cell culture, where structural integrity is preserved independently of polymer crosslinking [15]. We hypothesized that a hydrophobic gel employing such water‐immiscible ILs as the solvent, rather than a hydrogel, would enable reversible sol‐gel transitions even in contact with aqueous cell culture media. Building on this concept, we sought to design a polymer network that could reversibly assemble and disassemble under light while remaining confined within the IL phase. For this purpose, we selected azobenzene as a photoresponsive unit, because its reversible *trans‐cis* photoisomerization offers a well‐established means of modulating molecular polarity and intermolecular interactions [18]. In IL, azobenzene‐containing *N*‐isopropylacrylamide copolymers have been shown to exhibit either UCST‐type phase transitions depending on the solvent polarity and composition [19, 20, 21, 22, 23, 24]. We therefore designed the A block of an ABC triblock copolymer as a random copolymer of *N*‐isopropylacrylamide and 4‐phenylazophenyl acrylamide, P(AzoAm‐*r*‐NIPAm), enabling light‐controlled solubility in non‐cytotoxic ILs. The B block (PBuA) was chosen for its compatibility with ILs, and the C block (PSt) for its insolubility. The resulting ABC triblock copolymer, P(AzoAm‐*r*‐NIPAm)‐*b*‐PBuA‐*b*‐PSt, was combined with a solvent‐quality‐tunable blend [25, 26, 27, 28, 29] of non‐cytotoxic ILs, tri‐*n*‐butylmethylphosphonium trifluoromethylsulfonylimide ([P4,4,4,1][TFSI]) and tetra‐*n*‐octylphosphonium trifluoromethylsulfonylimide ([P8,8,8,8][TFSI]) (Figure 1). The photoresponsive A block undergoes reversible changes in solubility upon *trans–cis* isomerization of azobenzene, while IL blending allows fine adjustment of the upper critical solution temperature (UCST). Rheological measurements reveal that this ion gel exhibits light‐dependent gelation temperature windows and repeated crossings of tan *δ* = 1 at 52°C, consistent with a lifetime‐governed mechanism of transient network rearrangement. In addition, human mesenchymal stem cells (hMSCs) adhered to and spread on the gel surface, indicating the cytocompatibility of the ion gel at 37°C. Overall, this study demonstrates a new materials strategy that combines water‐immiscible ILs and photoreversible block copolymers to achieve reversible sol‐gel transitions in a closed system. Mechanical switching at cytocompatible temperatures has not yet been achieved, and strategies to shift the sol‐gel transition toward physiological temperature are discussed as future directions.

## 2 Results and Discussion

### 2.1 Design Rationale: Hierarchical Assembly in ILs and Light‐Programmable Solubility

The rationale for adopting an ABC triblock copolymer architecture rather than a conventional ABA design lies in the efficiency of network formation. ABA triblock copolymers often suffer from looped‐back chains and dangling ends, resulting in high critical gelation concentrations. In contrast, ABC triblock copolymer systems with mutually incompatible end blocks (A and C) suppress looping and favor bridging conformations, enabling stepwise, hierarchical assembly. Prior studies have demonstrated that such ABC architectures undergo micellization followed by secondary association, yielding gelation at relatively lower concentrations than ABA analogues [30, 31]. For example, gelation of ABC‐type triblock copolymers (PNIPAm‐*b*‐poly(ethylene oxide) (PEO)‐*b*‐poly(ethylene‐*alt*‐propylene)) has been reported at polymer concentrations below 1 wt. %, whereas ABA‐type analogues (PNIPAm‐*b*‐PEO‐*b*‐PNIPAm) typically require 5 wt. %–10 wt. % [30]. Similar reductions in percolation concentration for ABC architectures have been observed in different polymer systems [31, 32]. In line with these reports, the present ABC triblock ion gel forms a stable network at 15 wt. %, which is lower than the polymer concentration required in our previous ABA‐type ion gel system (20 wt. %) [21], highlighting the efficiency of the ABC design. Similar hierarchical sol‐gel transitions have also been observed in IL media [33], highlighting the generality of this approach. These precedents motivated our choice of an ABC design to establish efficient percolation in ILs.

To impart external control over self‐assembly, we incorporated azobenzene units into the thermoresponsive A block. Light was selected as the stimulus because it enables remote, non‐contact manner with high spatiotemporal precision, avoiding perturbations of cellular metabolism that are inevitable under thermal or chemical triggers. As a prerequisite, we confirmed that azobenzene exhibits reversible photochromism even in ILs: UV irradiation induces the *trans*‐to‐*cis* isomerization, which is reversed upon visible‐light exposure (Figures S1–S3). The absorption spectral changes are fully consistent with the well‐established behavior of azobenzene derivatives; a detailed discussion of the underlying photochromism is provided in the Supporting Information. Quantitative analysis revealed that the copolymer adopts nearly 100 % *trans* configuration under dark conditions, while UV irradiation converts approximately 80 % of the chromophores to the *cis* form, establishing a well‐defined and reproducible bistable photoisomerization ratio in the IL environment. This result validates the use of azobenzene photochromism as a reliable molecular trigger in IL environments, forming the basis of the present light‐programmable scaffold design.

### 2.2 Solubility of PNIPAm in ILs

As a first step toward designing the photoresponsive A block, we examined thermo‐sensitive PNIPAm homopolymer as the main component of the photoresponsive block. PNIPAm is one of the most widely studied water‐soluble polymers, known for its lower critical solution temperature (LCST)‐type phase transition at ∼32°C in aqueous solution. Interestingly, however, it has been reported that PNIPAm undergoes opposite upper critical solution temperature (UCST)‐type phase transitions in hydrophobic ILs containing anions such as TFSI [19, 20, 21, 22, 23, 24, 34, 35]. Given that the ILs targeted in this work also contain the TFSI anion, it was important to clarify the phase behavior of PNIPAm in these solvents. In principle, the phase transition temperature of a polymer can be shifted by copolymerization with more soluble or less soluble comonomers [36, 37] or by tuning the molecular weight [38]. Here, however, we adopted a simpler and more versatile strategy: blending two ILs with different solubilities for PNIPAm. Previous studies have shown that IL blends often allow nearly linear tuning of polymer compatibility with respect to composition [25, 26, 27, 28, 29].

**FIGURE 2:**
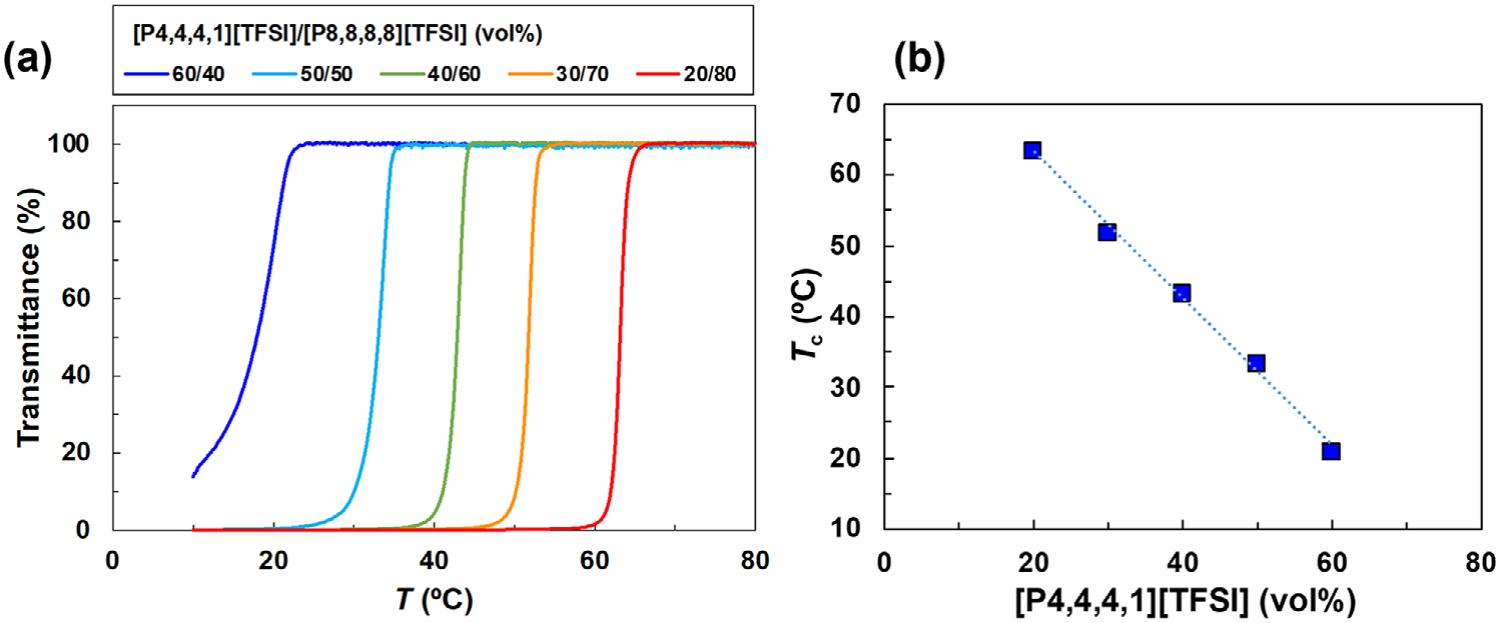
(a) Temperature dependence of transmittance for 2 w/v % PNIPAm solutions in [P4,4,4,1][TFSI]/[P8,8,8,8][TFSI] blends with different mixing ratios. Transmittance was monitored at 700 nm during cooling at 0.1°C min^−^^1^, and the cloud point temperature (*T*_c_) was defined as the inflection point of the transmittance curve. (b) Dependence of *T*_c_ on the volume fraction of [P4,4,4,1][TFSI] in IL blends. The dotted line represents a linear fit to the data, indicating that *T*_c_ can be continuously tuned by IL blending.

We therefore evaluated the solubility of well‐defined PNIPAm (*M*_n_ = 1.10 × 10^4^ g mol^−1^, *M*_w_/*M*_n_ = 1.26, Table S1) in ILs, [P4,4,4,1][TFSI] and [P8,8,8,8][TFSI], over the temperature range of 4°C–120°C (Table S2). PNIPAm was fully miscible in [P4,4,4,1][TFSI] throughout this range, whereas it exhibited a UCST‐type transition in [P8,8,8,8][TFSI], being insoluble below ∼100°C and soluble above it. By blending the two ILs, the transition temperature (*T*_c_) was continuously shifted: as the function of [P4,4,4,1][TFSI] increased, *T*_c_ decreased in an approximately linear fashion (Figure 2a,b). These results demonstrate that PNIPAm exhibits UCST‐type phase behavior in ILs and that the *T*_c_ can be widely tuned simply by adjusting the mixing ratio of [P4,4,4,1][TFSI] and [P8,8,8,8][TFSI]. This straightforward blending strategy provides a powerful and experimentally facile means of positioning the phase transition in the vicinity of biologically relevant temperatures.

### 2.3 Solubility of P(AzoAm‐*r*‐NIPAm) in ILs Under Light Illumination

**FIGURE 3:**
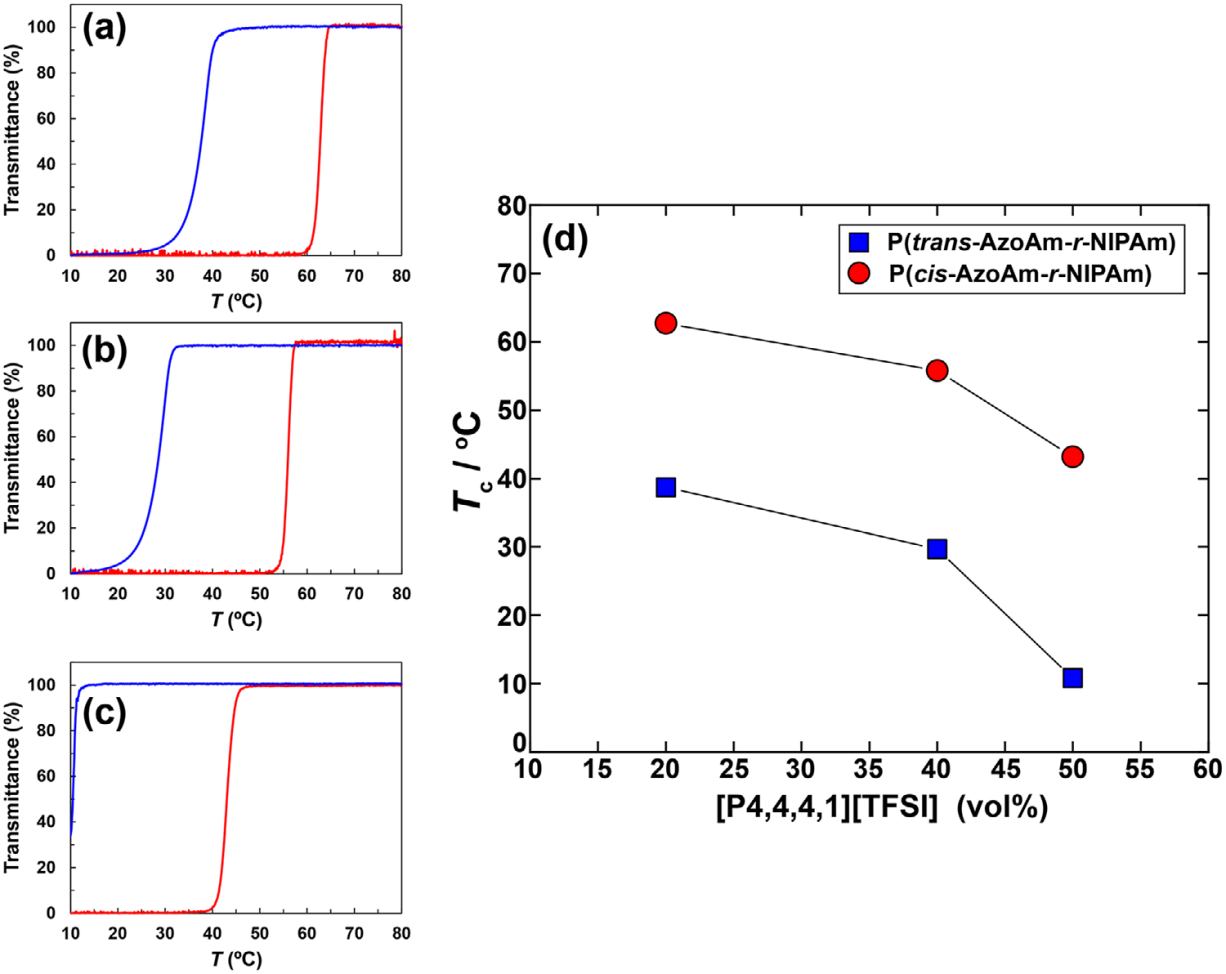
Temperature dependence of transmittance for 2 w/v % P(AzoAm‐*r*‐NIPAm) solutions in [P4,4,4,1][TFSI]/[P8,8,8,8][TFSI] blends of (a) 20/80, (b) 40/60, and (c) 50/50 vol % under UV irradiation at 365 nm (*cis*‐type, red lines) and in the dark (*trans*‐type, blue lines). Transmittance was monitored at 700 nm during cooling at 0.1°C min^−^^1^, and the cloud point temperature (*T*_c_) was defined as the inflection point of the curves. (d) Summary of *T*_c_ values as a function of [P4,4,4,1][TFSI] vol % in [P4,4,4,1][TFSI]/[P8,8,8,8][TFSI] blends, plotted separately for *cis*‐type (UV‐irradiated, red circle) and *trans*‐type (dark, blue square) states.

As a next step toward constructing the ABC triblock copolymer, we examined the phase behavior of the photoresponsive A block segment alone. A random copolymer of AzoAm and NIPAm, P(AzoAm‐*r*‐NIPAm), was synthesized via reversible addition fragmentation chain transfer (RAFT) copolymerization (Scheme S1, *M*_n_ = 1.38 × 10^4^ g mol^−^^1^, *M*_w_/*M*_n_ = 1.28; Table S1). The [AzoAm] content in the copolymer was 2.9 mol %. Turbidity measurements showed that P(AzoAm‐*r*‐NIPAm) undergoes a UCST‐type transition in IL blends. In [P4,4,4,1][TFSI]/[P8,8,8,8][TFSI] = 20/80 (vol/vol), *T*_c_ was 39°C in the *trans*‐type and 63°C in the *cis*‐type, giving a Δ*T*_c_ of 24°C (Figure 3a). This value is notably larger than Δ*T*_c_ typically reported for azobenzene‐containing polymers in aqueous systems [39], underscoring the high sensitivity of ILs to small structural perturbations. Such sensitivity can be partially rationalized from a thermodynamic perspective: our previous thermodynamic studies on the LCST‐type phase behavior of poly(benzyl methacrylate) in ILs revealed that both the enthalpy and entropy changes of mixing (Δ*H*_mix_ and Δ*S*_mix_) are negative but much smaller in magnitude than in aqueous systems [40]. Consequently, even slight changes in polymer or in solvent structure can induce large relative variations in Δ*H*_mix_ and/or Δ*S*_mix_, which in turn cause substantial shifts in the phase transition temperature. While the present system involves UCST‐type transitions and different polymer/IL combinations, and thus the same thermodynamic rationale may not apply strictly, this framework provides a plausible explanation for why Δ*T*_c_ values are unusually large in ILs compared with conventional aqueous media.

Increasing the fraction of [P4,4,4,1][TFSI] monotonically lowered *T*_c_ in both photoisomerization‐states (Figure 3b,c), and the dependence of *T*_c_ on composition is summarized in Figure 3d. The relative solubility of *cis*‐ and *trans*‐type polymers in ILs also revealed a striking contrast to previous observations in imidazolium ILs such as 1‐ethyl‐3‐methylimidazolium trifluoromethylsulfonylimide ([C_2_mim][TFSI]) and 1‐butyl‐3‐methylimidazolium hexafluorophosphate ([C_4_mim]PF_6_) [19, 20, 21, 22, 23, 24]. Here, the solvent polarity is discussed in terms of the *E*_T_(30) parameter, which represents the empirical solvent polarity scale (in kcal mol^−1^) derived from the solvatochromic shift of Reichardt's dye. In those relatively higher‐polarity ILs (*E*_T_(30): [C_2_mim][TFSI] = 52.2 kcal mol^−^^1^, [C_4_mim]PF_6_ = 52.4 kcal mol^−^^1^ [41]), the more polar *cis*‐azobenzene is preferentially solvated, resulting in higher LCST‐type *T*_c_ for the *cis*‐rich polymer [19, 20, 21, 22, 23, 24]. In contrast, the ILs used here are of lower polarity (*E*_T_(30): [P4,4,4,1][TFSI] = 46.6 kcal mol^−^^1^, [P8,8,8,8][TFSI] = 44.4 kcal mol^−^^1^ [15]), and the less polar *trans*‐rich polymer dissolves more readily, giving lower UCST‐type *T*_c_, meaning good solubility in the *trans*‐ than in the *cis*‐type. Such polarity‐dependent inversion of solubility preference is consistent with earlier studies on azobenzene‐ and spirobenzopyran‐containing copolymers in nonpolar organic solvents such as cyclohexane [42, 43, 44]. Together, these results establish that P(AzoAm‐*r*‐NIPAm) exhibits both a large, reliable Δ*T*_c_ and a polarity‐dependent inversion of *cis*/*trans* solubility in ILs, validating it as a robust light‐responsive segment for constructing photoreversible ion gels.

### 2.4 Photoreversible Sol‐Gel Transition and Viscoelastic Switching of the Non‐Cytotoxic Ion Gel

The ABC triblock copolymer, P(AzoAm‐*r*‐NIPAm)‐*b*‐PBuA‐*b*‐PSt, was synthesized via successive RAFT polymerizations (Scheme S2). Molecular weights and AzoAm feed compositions were determined by GPC and ^1^H NMR (Table S1), giving narrowly dispersed samples. In this architecture, the A block is the photoresponsive random copolymer P(AzoAm‐*r*‐NIPAm) (light‐tunable solubility), the B block (PBuA) is compatible with both [P4,4,4,1][TFSI] and [P8,8,8,8][TFSI], and the C block (PSt) is insoluble in these ILs. Hydrophobic B and C blocks were intentionally selected so that, even when the ion gel enters a sol‐like state, polymer chains remain confined to the IL phase and do not dissolve into the aqueous culture medium. We denote the triblock as ANBS, with subscripts indicating the AzoAm mol % in the A block. A_4.1_NBS and A_16.8_NBS correspond [AzoAm] = 4.1 mol % and [AzoAm] = 16.8 mol % containing in A block, respectively.

**FIGURE 4:**
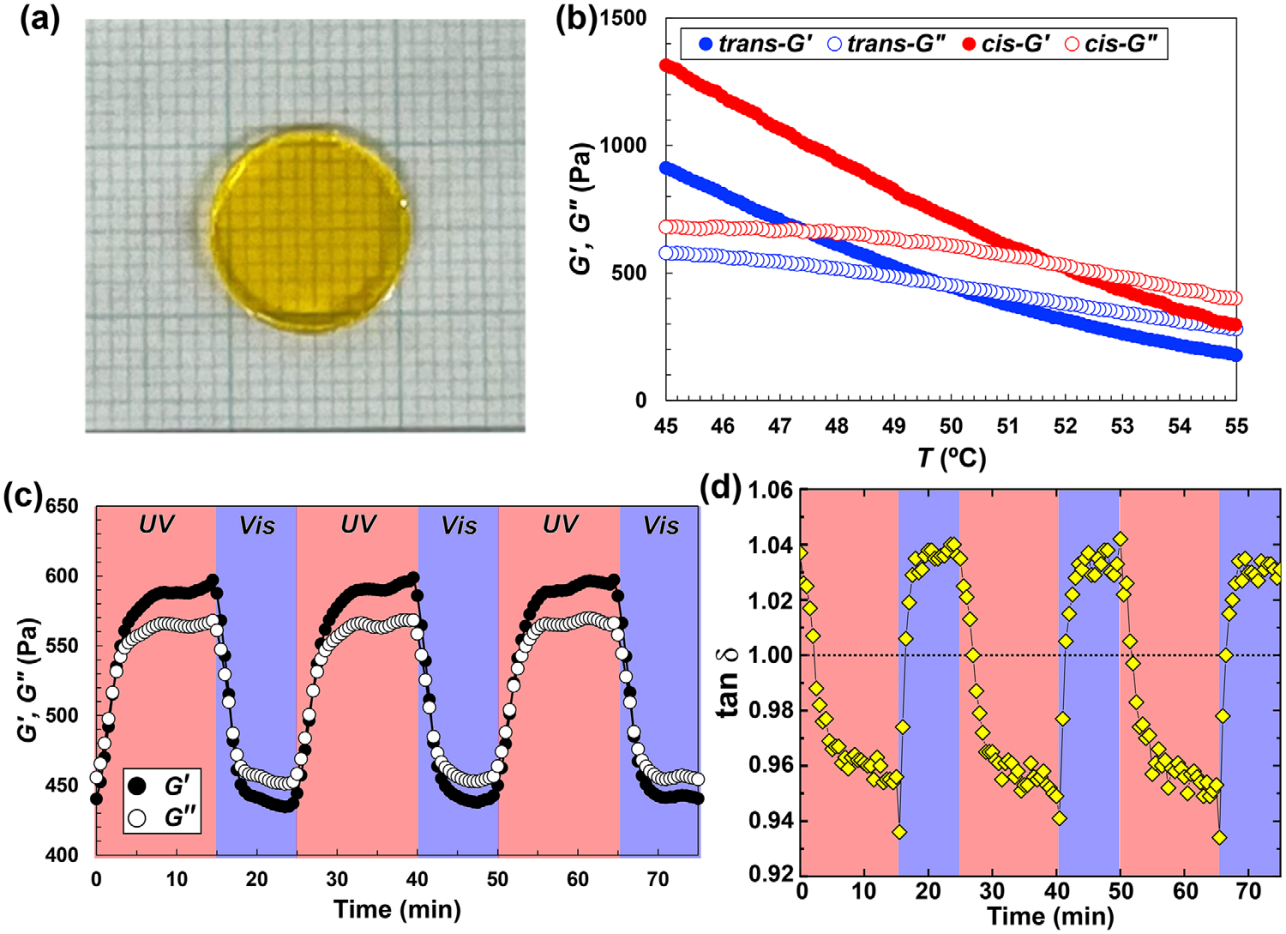
(a) Photograph of a transparent ion gel prepared from 15 wt. % A_4.1_NBS in an IL blend ([P4,4,4,1][TFSI]/[P8,8,8,8][TFSI] = 20/80 vol %) at room temperature. (b) Temperature dependence of storage (*G*′, solid symbols) and loss (*G*″, open symbols) moduli for 15 wt. % A_16.8_NBS ion gel at *ω* = 1 rad s^−^^1^ and *γ* = 1 % under visible (436 nm, blue) and UV (365 nm, red) illumination. The gelation temperature (*T*_gel_), defined as the crossover point *G*′ = *G*″, shifted from 50°C (vis) to 52°C (UV), demonstrating light‐dependent control of the sol‐gel transition. (c) Reversible sol–gel transitions at 52°C induced by alternating UV (15 min) and visible light (10 min) irradiation, showing reproducible modulation of *G*′ and *G*″ over three cycles at *ω* = 1 rad s^−^^1^. Notably, compared with the previous ABA‐type triblock design that required 20 wt. % polymer to achieve photoreversible gelation in ILs [21], the present ABC architecture enables stable gel formation at 15 wt. %, highlighting that hierarchical assembly of ABC triblocks improves the efficiency of network percolation. (d) Time‐dependent evolution of loss tangent (tan *δ*) at *ω* = 1 rad s^−^^1^ during the photo‐switching experiment shown in (c), explicitly demonstrating repeated crossings of tan *δ* = 1 under alternating light illumination. The dotted line indicates the sol‐gel boundary under observation frequency (tan *δ* = 1).

A transparent ion gel was obtained at room temperature by combining ANBS with an IL mixture ([P4,4,4,1][TFSI]/[P8,8,8,8][TFSI] = 20/80 vol %) via a co‐solvent evaporation method (Figure 4a). Temperature sweeps (*ω* = 1 rad s^−^^1^) on A_16.8_NBS under continuous illumination (UV = 365 nm, vis = 436 nm) revealed light‐dependent gelation windows (Figure 4b): under visible light (*trans*‐rich), *G*″ > *G*′ above 50°C and *G*′ > *G*″ below 50°C; under UV (*cis*‐rich), the rheological crossover (*G*′ = *G*″, operational *T*_gel_) shifted to 52°C. Thus, illumination toggles the liquid‐solid (gel) boundary by ∼2°C and drives tan *δ* across unity, consistent with an externally addressable sol‐gel transition.

Frequency sweeps (Figure S4a) further depict the mechanical states under visible light: at 70°C, terminal flow is observed (*G*′ ∼ *ω* ^2^, *G*″ ∼ *ω*); at 37°C, a gel‐like response persists (*G*′ > *G*″ over 0.1–100 rad s^−^^1^); and at 50°C the system approaches the rheological crossover characteristic of sol‐gel transitions [44, 45]. ABC‐type triblock copolymers, in which both terminal blocks exhibit LCST‐type phase transition behavior with different transition temperatures, have previously been shown to undergo stepwise self‐assembly from unimers to micelles and subsequently to polymer networks upon heating in ILs [33]. In the present system, although the molecular design differs in that the C block (polystyrene) is permanently immiscible with the ILs and the A block exhibits UCST‐type phase transition behavior, we consider that a related hierarchical assembly process occurs. Upon cooling, micellar cores formed by the C block are bridged through aggregation of the A blocks, leading to the formation of a polymer network.

In a transient‐network framework, the sol‐gel crossover frequency (*ω*_c_) is set by *ω*_c_ ∼ 1/*τ*, where *τ* is the exchange lifetime of A block aggregates. Under UV light irradiation (Figure S4b), *ω*_c_ at 50°C shifts from 10.6 s (under visible light) to 13.9 s (under UV light), indicating that A‐block aggregates act as more persistent crosslinking points under UV light. From the temperature‐sweep results at *ω* = 1 rad s^−^^1^ within 50–52°C, the A blocks behave as effective crosslinking points under UV light irradiation, whereas they disaggregate under visible light. Accordingly, under visible light, the micellar structure consists of C‐block cores with A and B blocks forming the corona, while under UV irradiation, a hierarchical micellar network is formed in which aggregated A blocks act as additional reversible junctions bridged by the B block (Figure 1).

Figure 4c demonstrates reversible switching at 52°C by alternating UV–vis light: *G*′ rises under UV and crosses *G*″ within ∼2 min, then drops under visible light and recrosses within ∼1 min, reproducibly over multiple cycles. To make this crossover more explicit, Figure 4d plots tan *δ* at *ω* = 1 rad s^−^^1^ against time under alternating illumination, showing repeated crossover across tan *δ* = 1, direct evidence that the material toggles between liquid‐like and solid‐like responses under light at a fixed probe frequency. Further cyclic durability and fatigue resistance will be examined in future studies, building on the chemical stability of ILs and the intrinsic reversibility of azobenzene photoisomerization.

To rationalize the repeated tan *δ* crossings observed under alternating illumination (Figure 4d), we interpret the viscoelastic response using a transient‐network framework, in which the sol‐gel crossover is governed by the lifetime of reversible junctions. At a fixed probing frequency (*ω* = 1 rad s^−^^1^), light primarily modulates the exchange lifetime (*τ*) of reversible junctions formed by A block aggregates, via changes in the effective *χ*‐parameter between the A block and the IL blend. Specifically, UV (*cis*‐rich) increases the incompatibility of the A block with the IL blend, lengthening τ and favoring a gel‐like state (*G*′ > *G*″), whereas visible light (*trans*‐rich) decreases *τ*, shifting the response toward a sol‐like state (*G*″ > *G*′). This lifetime‐driven switching is consistent with transient network analyses of ion gels, where *τ* ∼ *τ*_0_ exp(*αχN*) and *ω*_c_ ∼ 1/*τ* determine the liquid‐to‐solid (gel) crossover [46]. Here, *τ* is the terminal relaxation of the transient gel under given conditions, *τ*_0_ is the terminal relaxation of bulk A block, *α* is a constant of order unity, *χ* is the Flory–Huggins interaction parameter [47], and *N* is the degree of polymerization. The lifetime shift of the photoresponsive block provides a physically grounded explanation for the observed tan *δ* crossings near 50–52°C. The kinetics of the tan *δ* crossing are governed by multiple factors, including light penetration through the gel, the intrinsic photoisomerization rate of azobenzene (Figure S2), and the diffusion and aggregation of polymer chains within the IL matrix. In particular, the rate‐limiting step of the sol‐gel transition is expected to be governed by polymer diffusion and aggregation dynamics rather than by molecular photoisomerization alone. In ion gels, network formation involves collective rearrangement of polymer chains, and similar diffusion‐ and aggregation‐limited kinetics have been discussed in our previous studies on polymer phase transitions in ILs [19, 20, 21].

At 37°C, the ion gel remains gel‐like under both photo‐states, yet *G*′ and *G*″ modulate reversibly with light (Figure S5). This indicates that even below the rheological crossover, light irradiation can induce subtle but reversible changes in viscoelastic properties without any material loss to the aqueous phase. Such stability contrasts with conventional hydrogels, in which partial network disassembly typically results in polymer dissolution and irreversible softening. Therefore, while complete sol‐gel switching is not achieved at physiological temperature, the present IL‐based ion gel provides a mechanically stable yet optically tunable scaffold that can deliver small‐amplitude viscoelastic perturbations to cells.

The demonstration of tan *δ* changes here is at 50∼52°C. To shift the crossover toward 37°C, several routes are available within the current design possibility: increasing the [P4,4,4,1][TFSI] fraction in the IL blend to lower the UCST of the A block and thereby depress *T*_gel_; tuning the A block composition (AzoAm content or molecular weight) to adjust the photo‐solubility contrast and junction stability; optimizing polymer concentration and B/C block lengths to set the percolation threshold closer to physiological conditions. These approaches provide a practical roadmap toward physiological‐temperature operation of photoreversible ion gels.

### 2.5 Cell Culture on the Ion Gel

**FIGURE 5:**
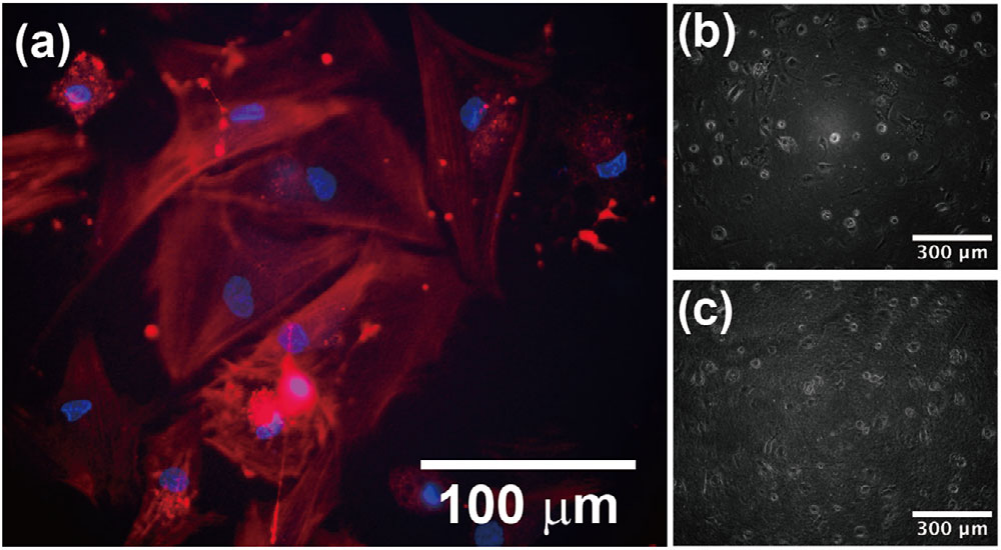
(a) Fluorescence image of hMSCs cultured on an A_4.1_NBS ion gel, stained for F‐actin (red) and nuclei (blue), showing that actin filaments were detectable and that the cells exhibited a spread morphology on the ion gel. (b,c) Microscopic images of cells cultured on A_16.8_NBS ion gels at 37°C that had been pre‐irradiated with (b) visible light (436 nm) or (c) UV light (365 nm) before cell seeding. In both cases, cells adhered and spread on the gel surfaces, indicating that photoisomerization of the A block and the associated viscoelastic changes do not impair the biocompatibility of the ion gel scaffold.

To assess cytocompatibility of the IL components, we performed an MTS assay using human mesenchymal stem cells (hMSCs). Both [P4,4,4,1][TFSI] and [P8,8,8,8][TFSI] showed cell viabilities comparable to the control without IL, whereas the typical imidazolium‐based IL [C_2_mim][TFSI] exhibited significant cytotoxicity (Figure S6). These results confirm that the non‐cytotoxic ILs employed in this study are suitable for cell culture. The polymer constituents of the ion gel, including PNIPAm, PBuA, PSt, and azobenzene derivatives, have also been widely reported to be cytocompatible. Therefore, the ion gel composed of these components is expected to provide a cytocompatible scaffold. We note that the present cell experiments focus on short‐term viability and adhesion, and that long‐term cytocompatibility and dynamic photo‐switching at physiological temperature will be addressed in future work. To investigate cell adhesion and spreading at the interface between the culture medium and the IL blends that constitute the major component of the gel, cells were seeded on IL blends with compositions of [P4,4,4,1][TFSI]/[P8,8,8,8][TFSI] = 50/50, 40/60, and 20/80 vol %. In all cases, cells adhered and spread without obvious cytotoxic effects (Figure S7). Encouraged by this result, we next cultured hMSCs directly on the A_4.1_NBS ion gel. Fluorescence staining of F‐actin revealed well‐aligned actin filaments extending across the cells, indicating robust adhesion and pronounced cytoskeletal organization on the ion gel surface (Figure 5a).

To further examine whether light irradiation prior to cell seeding affects cellular adhesion, cells were cultured on A_16.8_NBS ion gels that had been pre‐irradiated with either visible light (436 nm) or UV light (365 nm) (Figure 5b,c). In both cases, cells adhered to and spread on the gel surface, confirming that photoisomerization of the A block and the associated viscoelastic changes (Figure S5) do not compromise the biocompatibility of the scaffold. The cells exhibited similarly extended morphologies under both illumination conditions, consistent with stable focal adhesion and cytoskeletal organization irrespective of photoisomerization state. Quantitative image analysis further confirmed that cell spreading area and circularity showed no statistically significant differences between the UV–vis‐pre‐irradiated ion gels (Figure S15). These results indicate that the IL‐based ion gels maintain stable and non‐toxic interfaces for cell adhesion irrespective of photoirradiation history, providing a robust platform on which the unique photoreversible mechanical properties can be further explored in future mechanobiology studies.

## 3 Conclusion

We have established a non‐cytotoxic, photoreversible ion gel scaffold that crosses the rheological boundary (tan *δ* ∼ 1) under light, thereby toggling between liquid‐like and solid‐like responses, an operational mode long inaccessible to water‐swollen hydrogels. The material integrates an ABC triblock architecture (P(AzoAm‐*r*‐NIPAm)‐*b*‐PBuA‐*b*‐PSt) with a solvent quality tunable IL blend ([P4,4,4,1][TFSI]/[P8,8,8,8][TFSI]). The photoresponsive A block P(AzoAm‐*r*‐NIPAm) exhibits a large, compositional‐control Δ*T*_c_ in ILs, providing an effective molecular trigger for light‐programmed self‐assembly. In the ion gel, temperature sweeps and frequency spectra demonstrate light‐dependent gelation windows and the critical‐gel signature near the crossover. Time‐resolved switching confirms repeatable crossings of tan *δ* = 1 at a fixed probe frequency. A junction lifetime‐governed mechanism, where photoisomerization of AzoAm alters the effective *χ* of the A block, shifting the reversible‐junction lifetime and thus the sol‐gel crossover frequency, provides a physically grounded explanation of the observed viscoelastic toggling. Finally, hMSCs adhered to and spread on the ion gel, indicating biocompatibility and the ability to support organized cytoskeletal architecture.

Looking forward, a critical next step toward biological utility is to shift the crossover temperature toward 37°C by IL‐blend tuning, A block composition/molecular‐weight optimization, polymer‐concentration, and B/C‐block adjustments. This study proposes a fundamental design concept for dynamic yet structurally stable ion gel scaffolds. If reversible liquid‐solid (gel) mechanical cues can be applied to cells in situ in the future, this approach has the potential to open new avenues for next‐generation mechanobiology.

## 4 Experimental Section/Methods

### 4.1 Materials

All reagents were purchased from Fujifilm Wako Pure Chemical Corporation (Japan) and Tokyo Chemical Industry (Japan) unless otherwise stated. Lithium trifluoromethylsulfonylimide (Li[TFSI]) and 2,2’‐azobis(isobutyronitrile) (AIBN) were obtained from Kanto Chemical (Japan) and used as received. [P4,4,4,1][TFSI] was purchased from Kanto Chemical (Japan) and vacuum dried at 120°C overnight before use. NIPAm was purchased from KJ Chemicals Corporation (Japan) and purified by recrystallization using a toluene/hexane (1/20 by weight) mixture. Styrene and *n*‐butyl acrylate were obtained from Fujifilm Wako Pure Chemical Corporation (Japan) and Tokyo Chemical Industry (Japan), respectively, and were used after purification by passing them through aluminum oxide (90 active basic 0.063–0.200 mm, Merck, Germany). 2‐(Dodecylthiocarbonothioylthio)‐2‐methylpropionic acid and sulfo‐SANPAH were purchased from Sigma–Aldrich (USA) and Thermo Fisher Scientific, respectively, and used as received.

### 4.2 Synthesis of [P8,8,8,8][TFSI]

Briefly, [P8,8,8,8][TFSI] was obtained by the ion‐exchange reaction of the cation precursor and the corresponding lithium salt as an anion precursor, as previously reported. [15] Tetra‐*n*‐octylphosphonium bromide ([P8,8,8,8]Br) (10.0 g, 17.7 mmol) and a slightly excess (x 1.1 by mol) amount of Li[TFSI] (5.60 g, 19.5 mmol) were mixed in 30 mL of ethanol. The reaction was carried out overnight at 80°C. After the evaporation of ethanol, the reaction mixture was washed with water five times to remove any unreacted water‐miscible impurities. A clear and colorless liquid was obtained after vacuum drying overnight at 120°C and returned to atmospheric pressure with Ar flow.

### 4.3 Synthesis of AzoAm

AzoAm was synthesized according to the previous procedure [38]. Initially, *p*‐aminoazobenzene (9.86 g, 50.0 mmol) and triethylamine (9.06 mL, 65.0 mmol) were dissolved in diethyl ether (50 mL). To this solution, a mixture of acryloyl chloride (4.87 mL, 60.0 mmol) and diethyl ether (20 mL) was added dropwise using a Pasteur pipette at 0°C under flowing nitrogen gas. The reaction proceeded for 4 h at 0°C under a nitrogen atmosphere. The byproduct, triethylammonium chloride, was extracted by vacuum filtration. Diethyl ether was evaporated using a rotary evaporator, and the resulting product was dissolved in dichloromethane (DCM). The solution was then washed with deionized water to remove any remaining triethylammonium chloride, concentrated using a rotary evaporator, and recrystallized from ethanol. The obtained AzoAm was confirmed by ^1^H‐NMR (400 MHz, CDCl_3_) (Figure S8).

### 4.4 Polymerization of P(AzoAm‐*r*‐NIPAm)

NIPAm was copolymerized with AzoAm via reversible addition fragmentation chain transfer (RAFT) polymerization (Scheme S1). AzoAm (233 mg, 0.928 mmol), NIPAm (3.40 g, 30 mmol), CTA (96.3 mg, 0.264 mmol), and AIBN (8.6 mg, 0.052 mmol) were dissolved in 13 mL of 1,4‐dioxane at room temperature. The solution was purged with argon gas for 15 min to remove dissolved oxygen and then heated at 70°C for 24 h to undergo RAFT polymerization. The products were purified by reprecipitation from diisopropyl ether as a poor solvent. The obtained orange powder was reprecipitated with acetone as a good solvent and diisopropyl ether as a poor solvent and dried under vacuum at 60°C overnight.

Following RAFT polymerization and purification, the CTA residue attached to the polymer (P(AzoAm‐*r*‐NIPAm)‐CTA) terminus was removed as follows. P(AzoAm‐*r*‐NIPAm)‐CTA (1.60 g, 0.134 mmol) and AIBN (0.672 g, 4.09 mmol) were dissolved in 13.5 mL of 1,4‐dioxane at room temperature. The solution was deaerated with argon gas bubbling for 30 min and heated at 70°C for 22 h under an argon atmosphere. The products were purified by reprecipitation from hexane as a poor solvent. The collected powder was reprecipitated using acetone as a good solvent and hexane as a poor solvent, followed by drying under vacuum at 60°C overnight to obtain an orange powder.

The monomer composition of AzoAm was estimated from ^1^H‐NMR (400 MHz, CDCl_3_) by calculating the integrated intensity ratio between the peaks obtained for AzoAm and NIPAm (AzoAm: 7.4–8.0 ppm, 9H, Ar H; NIPAm: 3.8–4.2 ppm, 1H, CH) (Table S1 and Figure S9). The molecular weight and polydispersity index were determined by gel permeation chromatography (GPC) using DMF containing 10 mmol/L lithium bromide as the eluent (Table S1 and Figure S10). The columns (Showa Denko, Tokyo, Japan) were calibrated using poly(methyl methacrylate) as a molecular weight standard.

### 4.5 Polymerization of Polystyrene Chain Transfer Agent (PSt‐CTA)

The triblock copolymer was synthesized via RAFT polymerization (Scheme S2). Initially, St (22.6 g, 217 mmol), CTA (304 mg, 0.834 mmol), and AIBN (27.3 mg, 0.166 mmol) were mixed to obtain a homogenous solution. The solution was deaerated by bubbling with argon gas for 15 min. The RAFT polymerization was performed at 70°C for 4 h. The products were purified by reprecipitation with acetone as a good solvent and diisopropyl ether as a poor solvent. The pale yellow powder was obtained and dried overnight under vacuum at 60°C. The obtained PSt‐CTA was confirmed by ^1^H‐NMR (400 MHz, CDCl_3_) (Figure S11). The molecular weight and polydispersity index were determined by GPC (Table S1 and Figure S14). Details of the GPC measurement are described above.

### 4.6 Polymerization of PSt‐*b*‐PBuA Chain Transfer Agent (PSt‐*b*‐PBuA‐CTA)

Polymerization and purification of PSt macroinitiator were followed by the synthesis of PSt‐*b*‐PBuA‐CTA (Scheme S2). BuA (6.74 g, 52.6 mmol), PSt‐CTA (1.00 g, 0.157 mmol), and AIBN (5.3 mg, 0.0323 mmol) were dissolved in 2 mL of 1,4‐dioxane at room temperature. The solution was purged with argon gas for 30 min to remove dissolved oxygen and then heated at 70°C for 4 h to undergo RAFT polymerization. The products were purified by reprecipitation from hexane as a poor solvent. The obtained sticky polymer was reprecipitated with acetone as a good solvent and hexane as a poor solvent and dried under vacuum at 60°C overnight. The obtained PSt‐*b*‐PBuA ‐CTA was confirmed by ^1^H‐NMR (400 MHz, CDCl_3_) (Figure S12). The molecular weight and polydispersity index were determined by GPC (Table S1 and Figure S14). Details of the GPC measurement are described above.

### 4.7 Polymerization of ANBS

Representative procedures for A_16.8_NBS (AzoAm/NIPAm = 16.8/93.2) are described as follows (Scheme S2). AzoAm (212 mg, 0.844 mmol), NIPAm (973 mg, 8.60 mmol), and PSt‐*b*‐PBuA‐CTA (1.03 g, 0.0269 mmol) were dissolved in 1,4‐dioxane (3.5 mL) at room temperature. AIBN (1.0 mg, 6.1 µmol) was separately dissolved in 1,4‐dioxane (500 µL) in another round‐bottom flask. The AIBN solution (435 µL) was then added to the monomer and macroinitiator solutions. The solution was deaerated with argon gas bubbling for 30 min. The RAFT polymerization was performed at 70°C for 49 h. The products were purified by reprecipitation from hexane as a poor solvent. The obtained sticky polymer was reprecipitated using acetone as a good solvent and hexane as a poor solvent, followed by reprecipitation using acetone as a good solvent and a mixture of ethanol/water (90/10 vol %) as a poor solvent. The collected polymer was dried overnight under vacuum at 60°C.

Subsequently, the dodecyl trithiocarbonate residue derived from CTA attached to the copolymer (CTA‐ANBS) terminus was removed following the same procedure described above. ANBS‐CTA (1.15 g, 0.0324 mmol) and AIBN (535 mg, 3.26 mmol) were dissolved in 15 mL of 1,4‐dioxane at room temperature. The solution was deaerated with argon gas bubbling for 30 min. The cleavage reaction was performed at 70°C for 19 h under an argon atmosphere. The products were purified by reprecipitation from hexane as a poor solvent. The collected sticky polymer was reprecipitated with acetone as a good solvent and hexane as a poor solvent and dried under vacuum at 60°C overnight to obtain an orange sticky solid.

The monomer composition of AzoAm is estimated from ^1^H‐NMR (400 MHz, CDCl_3_) by calculating the integrated intensity ratio between the peaks obtained for AzoAm and NIPAm (AzoAm: 7.3–8.0 ppm, 9H, Ar H; NIPAm: 1.0–1.2 ppm, 6H, CH_3_) (Table S1 and Figure S13). The molecular weight and polydispersity index were determined by GPC (Table S1 and Figure S14). Details of the GPC measurement are described above.

### 4.8 Preparation of PNIPAm and P(AzoAm‐*r*‐NIPAm) Solution in ILs

Polymer solutions were obtained by the cosolvent evaporation method. PNIPAm or P(AzoAm‐*r*‐NIPAm) was first dissolved in DCM. Appropriate amounts of [P4,4,4,1][TFSI] and [P8,8,8,8][TFSI] were added to the homogeneous polymer solution to obtain a final polymer concentration of 2 w/v %. After mixing well, the volatile DCM was evaporated by heating the solution at 110°C under reduced pressure overnight.

### 4.9 Preparation of an Ion Gel

The ANBS triblock copolymer was dissolved in a mixture of [P4,4,4,1][TFSI], [P8,8,8,8][TFSI] (20:80 vol %) and DCM. The weight ratio of the ANBS and IL mixture was 15:75 wt. %. After thorough mixing, DCM was evaporated by heating the solution at 85°C under reduced pressure overnight.

### 4.10 Turbidity Measurement

The temperature‐dependent phase separation behaviors of PNIPAm and P(AzoAm‐*r*‐NIPAm) were examined by performing transmittance measurements using a UV–vis spectrophotometer (UV–vis; V‐770, JASCO, Japan) with a temperature controllable unit (ETCS‐761, JASCO, Japan). The polymer solution (2 w/v %) was prepared via the cosolvent evaporation method, followed by cooling at room temperature in the dark for 24 h. The solution was stirred and heated at 80°C for 20 min either in the dark or under UV irradiation (50 mW), and then transferred to a quartz cell. Photoirradiation was performed using a spotlight source (LC8, HAMAMATSU Photonics, Japan) and a band‐pass filter (Edmund Optics, USA). The solution was heated again at 80°C for 10 min, followed by transmittance measurement at 700 nm while cooling the solution at 0.1°C min^−1^. Throughout the measurements, the polymer solution was stirred at 130 rpm and kept in the dark or constantly exposed to UV light from above. The cloud point was determined as the temperature at the inflection point of the transmittance curve.

### 4.11 Rheological Measurement

The rheology of the ion gel was investigated using an Anton Paar Physica MCR 102 instrument (Anton Paar, Austria) with a standard cell for UV irradiation (P‐PTD200/GL). A parallel plate geometry with a diameter of 12 mm and a gap spacing of approximately 0.1 mm was used for the measurement. UV light (365 nm, 1.5 mW) or visible light (436 nm, 0.97 mW) was used to illuminate the sample from the bottom side using a spotlight source (LC8, HAMAMATSU Photonics, Japan) over 1 h at 80°C to reach a photostationary state prior to measurement. Temperature scanning measurements were performed with a strain of *γ* = 1 %, a frequency of *ω* = 1 rad s^−1^, and a cooling rate of 0.1°C min^−1^ in the range of 80–10°C under continuous light irradiation.

The photoswitching measurements were also performed at the same strain and frequency at 52°C. Before measurement, the ANBS ion gel was illuminated under visible light (436 nm, 0.97 mW) from the bottom side and was maintained at 52°C for over 30 min.

### 4.12 Culturing Cells on IL Mixtures and an Ion Gel

A solution of fibronectin (2 mL, 6 µg/mL) in pH 7.4 phosphate‐buffered saline (PBS) was covered over an IL blend followed by incubation in 5 % CO_2_ at 37°C for 2 h. The upper fibronectin solution was washed six times with 1 mL of culture medium. Madin Darby Canine Kidney (MDCK) were seeded onto the IL blend with a density of 3.7 × 10^4^ cells/cm^2^. After incubation in 5 % CO_2_ at 37°C for 23 h, the cells on the IL mixtures were observed under an Olympus IX89 microscope. MDCK on the IL blend ([P4,4,4,1][TFSI]/[P8,8,8,8][TFSI] = 20/80 vol %) were stained with Hoechst 33342 (Life Technologies, 2548w, dilution of 1:1000) for 10 min to visualize the nucleus. Finally, Cells on the IL mixture were soaked in PBS and observed using an Olympus BX51 microscope.

hMSCs (bone marrow) (PromoCell) were cultured in basal growth medium supplemented with MSC growth supplements, L‐glutamine, gentamycin sulfate (30 mg mL^−1^), and amphotericin‐B (15 ng mL^−1^) (Lonza) according to the manufacturer's instructions. The cells were incubated in 5 % CO_2_ at 37°C. Cells above passage five were used. Before cell culture, ANBS ion gels were coated with collagen. The ion gels were immersed in a 4‐(2‐hydroxyethyl)‐1‐piperazineethanesulfonic acid (HEPES) buffer solution (50 mM, pH 8.5) containing 1 mg/mL sulfo‐SANPAH and exposed to UV light (10 J cm^−2^). The gels were washed three times with PBS, immersed in a HEPES buffer solution containing 0.1 mg/mL collagen type I (Corning, USA), and incubated overnight at 4°C. The collagen‐coated ion gels were washed three times with the culture medium. hMSCs were seeded on the gel with a density of 1.8 × 10^4^ cells cm^−2^. After incubation in 5 % CO_2_ at 37°C for 24 h, hMSCs on the ion gel were stained with phalloidin Alexa Fluor 568 (Life Technologies, A12380, dilution of 1:200) and Hoechst 33342 (Life Technologies, 2548w, dilution of 1:1000) for 10 min to stain *F*‐actin and nuclei, respectively. hMSCs on the ion gel were soaked in PBS and observed under an Olympus BX51 microscope. Other collagen‐coated ion gels were washed three times with PBS and irradiated with UV light (365 nm, 1.5 mW) for 30 min and visible light (436 nm, 0.97 mW) for 20 min using a spotlight source (LC8, HAMAMATSU Photonics, Japan) through a bandpass filter (Edmund Optics, USA). After replacing upper PBS with culture medium, hMSCs were seeded on the ion gels with a density of 1.0 × 10^4^ cells cm^−2^, followed by incubation in 5 % CO_2_ at 37°C for 6 h. Subsequently, the cells were observed under an Olympus IX89 microscope.

### 4.13 MTS Assay

hMSCs were seeded in 96‐well plates at 6.0 × 10^3^ cells/well and allowed to attach for 24 h. The culture medium was then replaced with [C_2_mim][TFSI], [P4,4,4,1][TFSI], or [P8,8,8,8][TFSI] saturated medium, followed by incubation for 24 h at 37°C and 5 % CO_2_. Cell viability was evaluated using an MTS ([3‐(4,5‐dimethylthiazol‐2‐yl)‐5‐(3‐carboxymethoxyphenyl)‐2‐(4‐sulfophenyl)‐2*H*‐tetrazolium]) assay according to the manufacturer's instructions after 24 h of culturing. The absorbance obtained from the MTS assay was defined as cell viability, which is the ratio of the absorbance of each sample divided by the absorbance of the culture medium in the absence of ILs.

## Funding

Grant‐in‐Aid for JSPS Fellows (22KJ010202 to A.S.) and JSPS KAKENHI grants (23H02030 to T. U.; and 23K17481 to J. N.).

## Conflicts of Interest

The authors declare no conflicts of interest.

## Supporting information




**Supporting File**: marc70211‐sup‐0001‐SuppMat.docx.

## Data Availability

The data that support the findings of this study are available on request from the corresponding author. The data are not publicly available due to privacy or ethical restrictions.
