# Supplementary material for: Toward Dynamic Liquid Cell Scaffold: Photoreversible Ion Gels Exhibiting Light‐Induced Sol‐Gel Transitions
Source: Macromol Rapid Commun. 2026 Jan 28;47(7):e00909. doi: 10.1002/marc.202500909 (PMC13047471; doi:10.1002/marc.202500909)
Supplement: Supplementary file 1 — Supporting File: marc70211‐sup‐0001‐SuppMat.docx. [file MARC-47-e00909-s001.docx]

Supporting Information

Toward Dynamic Cell Scaffold: Photoreversible Ion Gels Exhibiting Light-Induced Sol-Gel Transitions

Aya Saruwatari^†^, Yuji Kamiyama, Ryota Tamate, Jun Nakanishi, and Takeshi Ueki*

A. Saruwatari, Y. Kamiyama, R. Tamate, J. Nakanishi, T. Ueki

Research Center for Macromolecules and Biomaterials, National Institute for Materials Science, 1-1 Namiki, Tsukuba, Ibaraki 305-0044, Japan
E-mail: UEKI.Takeshi@nims.go.jp

A. Saruwatari, T. Ueki
Graduate School of Life Science, Hokkaido University, Kita 10, Nishi 8, Kita-ku, Sapporo, Hokkaido 060-0810, Japan

J. Nakanishi

Graduate School of Advanced Science and Engineering, Waseda University, 3-4-1 Okubo, Shinjuku-ku, Tokyo 169-8555, Japan

Graduate School of Advanced Engineering, Tokyo University of Science, 6-3-1 Niijuku, Katsushika-ku, Tokyo 125-8585, Japan

^†^Present address: Graduate School of Engineering, The University of Tokyo, Tokyo 113-8654, Japan

Keywords: photoreversible gelation, ionic liquids, ion gels, ABC-type triblock copolymers, azobenzene, mechanobiology, stimuli responsive materials

*Polymerization of PNIPAm*: PNIPAm was synthesized via RAFT polymerization. NIPAm (3.40 g, 30 mmol), 2-(dodecylthiocarbonothioylthio)-2-methylpropionic acid as a charge transfer agent (CTA) (124 mg, 0.340 mmol), and AIBN (11.1 mg, 0.0676 mmol) were dissolved in 1,4-dioxane (12 mL) at 25 °C. The solution was deaerated by bubbling with argon gas for 30 min. The RAFT polymerization was performed at 70 °C for 24 h. The products were purified by reprecipitation using hexane as a poor solvent. The collected powder was reprecipitated with acetone as a good solvent and diisopropyl ether as a poor solvent and dried under vacuum at 60 °C overnight to obtain a pale-yellow powder.

Subsequently, the dodecyl trithiocarbonate residue derived from CTA attached to the polymer (PNIPAm-CTA) terminus was removed. PNIPAm-CTA (2.00 g, 0.217 mmol) and AIBN (1.07 g, 6.52 mmol) were dissolved in 23 mL of 1,4-dioxane at room temperature. The solution was deaerated with argon gas bubbling for 30 min. The cleavage reaction was carried out at 70 °C for 22 h under an argon atmosphere. The products were purified by reprecipitation from hexane as a poor solvent. The collected powder was reprecipitated with acetone as a good solvent and hexane as a poor solvent and dried under vacuum at 60 °C overnight to obtain a white powder.

The molecular weight and polydispersity index were determined by gel permeation chromatography (GPC) using DMF containing 10 mmol/L lithium bromide as the eluent (**Table S1**, **Figure S10**). The columns (Showa Denko, Tokyo, Japan) were calibrated using poly(methyl methacrylate) as a molecular weight standard.

*Photochromism and thermal isomerization reaction of AzoAm in NCILs*: To investigate the photoisomerization of AzoAm monomer solutions in [P4,4,4,1][TFSI] and [P8,8,8,8][TFSI], 0.001 wt% AzoAm solutions in [P4,4,4,1][TFSI] and [P8,8,8,8][TFSI] were prepared. The absorbance spectra were measured using a UV–Vis spectrophotometer (UV-2600, Shimadzu, Japan). Before the measurements, UV light (365 nm, 0.48 mW) was irradiated using a mercury lamp (SX-UI 251HQ, USHIO, Japan) for 1 h. The time course of the absorbance spectra was traced under visible light (436 nm, 0.37 mW) for 30 min, followed by the same investigation under UV light. These cyclic irradiations were repeated a total of three times. The wavelength of the irradiated light was switched using bandpass filters (Edmund Optics, USA). A heat-absorbing filter (Edmund Optics, USA) was used to cut the heat generated by the mercury lamp.

**Table S1.** Characterization results for polymers.

| Polymer | | *M*_n_  [kDa] | *M*_w_ /*M*_n_  [-] | [AzoAm]/[NIPAm] [mol%] |
| --- | --- | --- | --- | --- |
| PNIPAm | | 11.0 | 1.26 | — |
| P(AzoAm-*r*-NIPAm) | | 13.8 | 1.28 | 2.9 |
| PSt-CTA | | 6.37 | 1.20 | — |
| PSt-*b*-PBuA-*b*-CTA | | 6.37–32.0 | 1.32 | — |
| P(AzoAm-*r*-NIPAm)-*b*-PBuA-*b*-PSt | A_4.1_NBS | 6.37–32.0–6.77 | 1.40 | 4.1 |
|  | A_16.8_NBS | 6.37–32.2–17.7 | 1.23 | 16.8 |

Table S2. Results of solubility tests for 2 w/v% PNIPAm in [P2,2,2,5][TFSI], [P4,4,4,1][TFSI] and [P8,8,8,8][TFSI] as solvents. Solutions were obtained using the cosolvent evaporation method, and transparency was evaluated visually. Yes: Transparent, homogeneous mixture with a range from 4 to 120 °C. No: Turbid phase separation within a range from 4 to 120 °C. UCST: UCST type phase transition within a range from 4 to 120 °C.

|  | [P2,2,2,5][TFSI] | [P4,4,4,1][TFSI] | [P8,8,8,8][TFSI] |
| --- | --- | --- | --- |
| PNIPAm | Yes | Yes | UCST  (~100 ºC) |

**Scheme S1.** Synthesis procedure of P(AzoAm-*r*-NIPAm).

**Photoisomerization reaction of AzoAm in NCILs**

To induce viscoelastic changes in response to light stimulation, the aggregation of terminal blocks was designed to be controlled by light. In addition to thermoresponsive phase transitions, azobenzene, whose solubility in solvents changes depending on its photoisomerization state, was used. By copolymerizing NIPAm with a monomer bearing azobenzene pendant groups, the solubility of the copolymer can be expected to change depending on the photoisomerization state of the azobenzene moiety.

AzoAm was synthesized to copolymerize NIPAm with azobenzene monomer. Before copolymerization, the reversibility of the photoisomerization of AzoAm in the NCILs was examined. In [P4,4,4,1][TFSI] and [P8,8,8,8][TFSI], similar to azobenzene chromophores in other molecular liquids,^[36, 39, 48]^ the peak corresponding to the π–π* transition of the *trans-*isomer decreased under 365 nm irradiation, whereas the absorption based on the n-π* transition of the *cis*-isomer increased. Conversely, under 436 nm irradiation, the peak of the n-π* transition corresponding to the *cis*-isomer decreased and the peak of the π–π* transition corresponding to the *trans*-isomer increased, returning to the original state (**Figure S1**). When 365 and 436 nm irradiations are applied alternatively for 30 min each, the absorbance of peaks in the UV region increases and decreases reversibly (**Figure S2**). Consequently, a reversible photoisomerization reaction between *cis-* and *trans*-states was confirmed.

**Figure S1.** Transition of absorbance spectra at 37 °C for 0.001 wt% AzoAm solution in [P4,4,4,1][TFSI] under 436 nm (0.37 mW) (a) and 365 nm (0.48 mW) (b), and for 0.001 wt% AzoAm solution in [P8,8,8,8][TFSI] under 436 nm (0.37 mW) (c), and 365 nm (0.48 mW) (d).


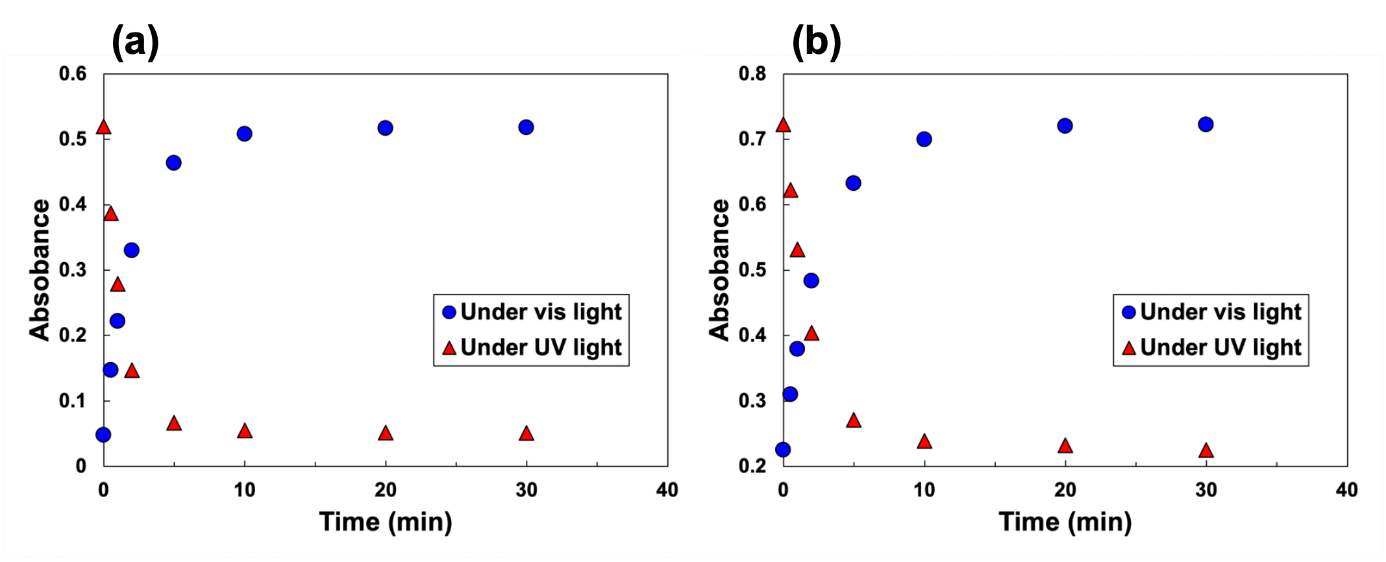


**Figure S2.** Transition of absorbance at 360 nm at 37 °C for 0.001 wt% AzoAm solution in [P4,4,4,1][TFSI] (a) and in [P8,8,8,8][TFSI] (b) under 436 nm (0.37 mW) and 365 nm (0.48 mW) irradiation.

**Figure S3.** Cyclic changes of absorbance at 360 nm for 0.001 wt% AzoAm solution in [P4,4,4,1][TFSI] (a) and [P8,8,8,8][TFSI] (b) with alternating visible (436 nm) and UV (365 nm) light for 30 min each.

**Scheme S2.** Synthesis procedure of ANBS.


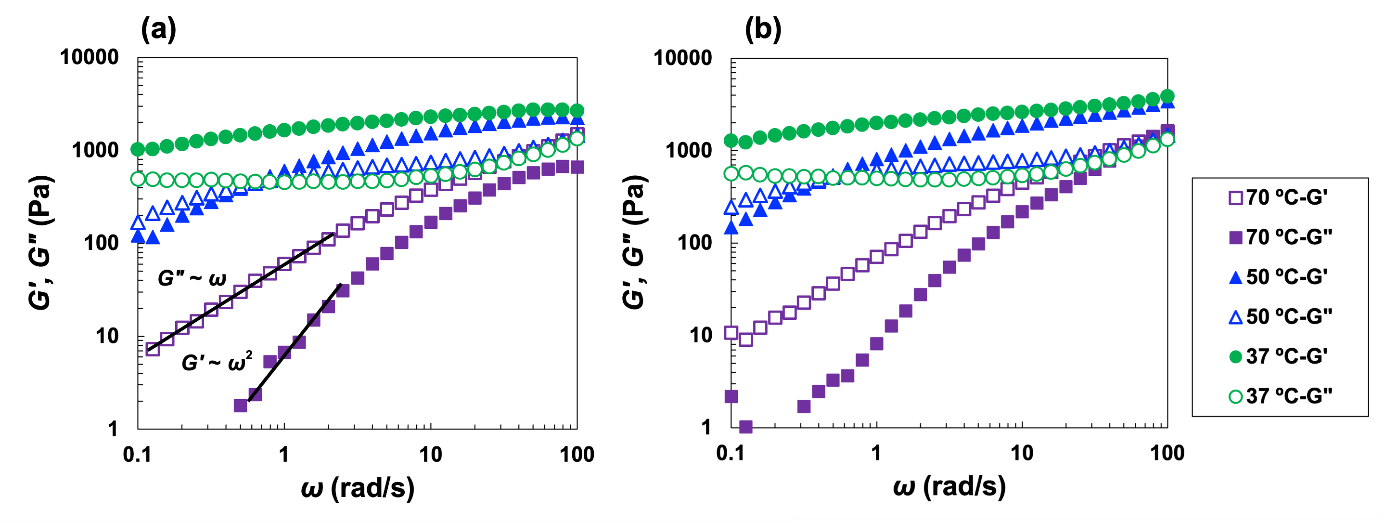


**Figure S4.** Variation of dynamic storage moduli (*Gʹ*, solid symbols) and loss moduli (*G″*, open symbols) of 15 wt% (a) *trans*-A_16.8_NBS and (b) *cis*-A_16.8_NBS in NCIL blend of [P4,4,4,1][TFSI]/[P8,8,8,8][TFSI] = 20/80 vol% %) as a function of frequency at 70 °C (purple squares), 50 °C (blue triangles), and 37 °C (green circles) at strain of *γ* = 1%.


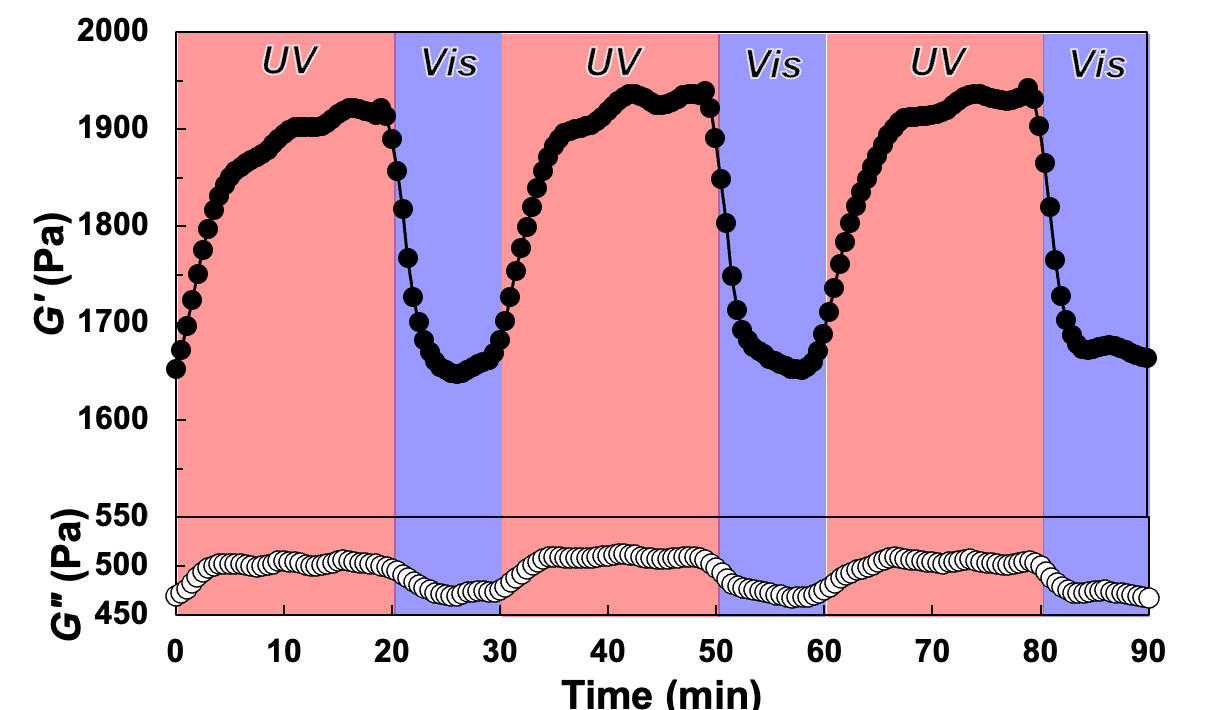


**Figure S5.** Reversible viscoelastic changes of A_16.8_NBS ion gel by alternately switching between UV and visible light irradiation in 20 and 10 min at 37 °C at a frequency of *ω* = 1 rad s^−1^ and a strain of *γ* = 1%.


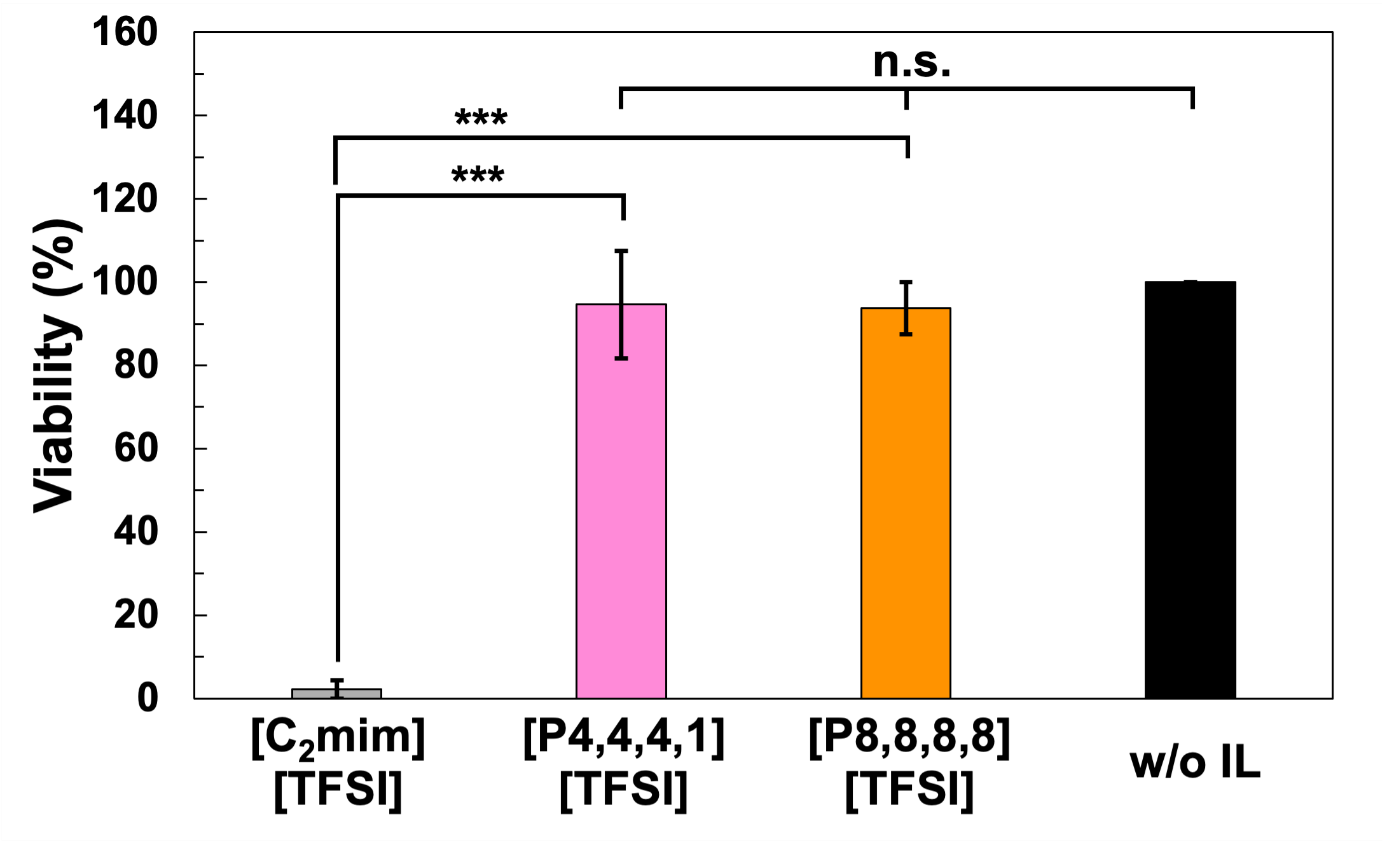


**Figure S6.** Cytotoxicity tests of hMSCs after culturing 24 h in the presence of 1-ethyl-3-methylimidazolium trifluoromethylsulfonylimide ([C_2_mim][TFSI]), [P4,4,4,1][TFSI] and [P8,8,8,8][TFSI] using the MTS assay. ****p* < 0.05 (two-tailed Student’s t test)

**Figure S7.** Microscopic observations of MDCK at liquid–liquid interfaces between culture medium and NCIL blends of [P4,4,4,1][TFSI]/[P8,8,8,8][TFSI] = 50/50 vol% (a), 40/60 vol% (b), and 20/80 vol% (c). (d) Fluorescence-stained image for nuclear of hMSC, acquired at same area as (c).

**Figure S8.** ^1^H-NMR spectra for AzoAm in chloroform-*d* (CDCl_3_).

**Figure S9.** ^1^H-NMR spectra for P(AzoAm-*r*-NIPAm) in chloroform-*d* (CDCl_3_).

**Figure S10.** Gel permeation chromatography (GPC) curves of PNIPAm (black) and P(AzoAm-*r*-NIPAm) (red).

**Figure S11.** ^1^H-NMR spectra for polystyrene (PSt)-charge transfer agent (CTA) in chloroform-d (CDCl_3_).

**Figure S12.** ^1^H-NMR spectra for PSt-*b*-poly(*n*-butyl acrylate) (PBuA)-CTA in CDCl_3_.

**Figure S13.** ^1^H-NMR spectra for SBNA_16.8_ in CDCl_3_.

**Figure S14.** GPC curves of PSt-CTA (black), PSt-*b*-PBuA-CTA (green), A_4.1_NBS(pink), and A_16.8_NBS (red).


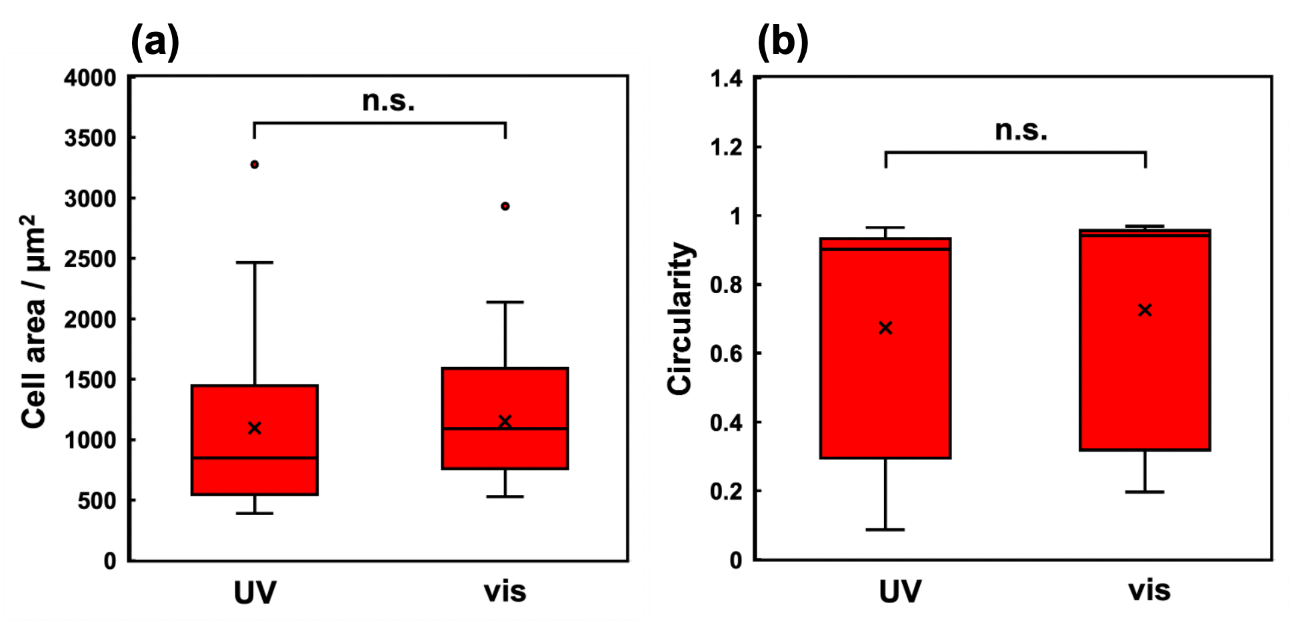


**Figure S15.** Quantitative analysis of cell morphology on A_16.8_NBS ion gels pre-irradiated with UV (365 nm) or visible light (436 nm): (a) cell spreading are and (b) circularity. No statistically significant differences were observed (*p* > 0.05, Student’s t-test)
